# Diminished Systemic and Mycobacterial Antigen Specific Anti-microbial Peptide Responses in Low Body Mass Index–Latent Tuberculosis Co-morbidity

**DOI:** 10.3389/fcimb.2020.00165

**Published:** 2020-04-28

**Authors:** Anuradha Rajamanickam, Saravanan Munisankar, Chandra Kumar Dolla, Subash Babu

**Affiliations:** ^1^National Institute of Health-NIRT-International Center for Excellence in Research, Chennai, India; ^2^Department of Epidemiology, National Institute for Research in Tuberculosis, Chennai, India; ^3^Laboratory of Parasitic Diseases, National Institute of Allergy and Infectious Diseases, National Institutes of Health, Bethesda, MD, United States

**Keywords:** low BMI, latent tuberculosis, anti-microbial peptides, HNP1-3, granulysin, HBD-2, cathelicidin

## Abstract

Low body mass index (BMI) is a risk factor for progression from latent *Mycobacterium tuberculosis* infection to active tuberculosis (TB) disease. Anti-microbial peptides (AMPs) are multifunctional molecules that play a crucial role in the mammalian host innate defense mechanism. AMPs have been shown to have an important role in host immunity to TB infection. The association of antimicrobial peptides with low BMI–latent tuberculosis (LTBI) co-morbidity has not been explored. To study the association of AMPs with LTBI-BMI, we examined the systemic, baseline, and mycobacterial antigen stimulated levels of human neutrophil peptides 1–3, (HNP1-3), granulysin, human beta defensin–2 (HBD-2), and cathelicidin (LL-37) in individuals with LTBI and low BMI (LBMI) and compared them with individuals with LTBI and normal BMI (NBMI). LBMI was characterized by diminished systemic levels of HNP1-3, granulysin, HBD-2 and cathelicidin in comparison with NBMI. Similarly, LBMI was also characterized by diminished unstimulated levels of HNP1-3 and granulysin and diminished mycobacterial antigen stimulated levels of HNP1-3, granulysin, and HBD-2. In addition, certain AMPs exhibited a positive correlation with BMI. Our data, therefore, demonstrates that coexistent LBMI in LTBI is characterized by the diminished levels of HNP1-3, granulysin, HBD-2, and cathelicidin, thereby potentially increasing the risk of progression to active TB.

## Introduction

Low BMI and tuberculosis are closely interlinked (Semba et al., 2010; Padmapriyadarsini et al., 2016). It is essential to decipher how these two problems tend to interact with each other. Both the innate and adaptive immune systems play a vital role in nutritional immunology (Maggini et al., 2018). Low BMI influences both the innate and adaptive immunity of an individual, making them more susceptible to infections (Rytter et al., 2014; Bourke et al., 2016; Chandrasekaran et al., 2017). Human and animal studies show that nutrition insufficiency leads to impaired cell mediated and humoral immune responses, which in turn affects the ability of an individual to fight against *Mycobacterium tuberculosis* (*M.tb)* infection or disease (Chan et al., 1996; Dai et al., 1998; Mainali and McMurray, 1998). In summary, it is thought that low BMI induces detrimental effects on many features of host immune responses against mycobacterial infection.

Anti-microbial peptides (AMPs) are multi-functional molecules, also known as cationic host defense peptides (Brown and Hancock, 2006). Most AMPs are comparatively short, generally comprising of 10–50 amino acids of an amphipathic nature and have a broad-spectrum of activity (Nguyen et al., 2011). AMPs are believed to be the first line of the innate immune defense and play a crucial role in the host defense mechanism against bacteria, viruses, and fungi (Zasloff, 2002; Schauber and Gallo, 2008; Hancock et al., 2016). Studies have shown that dysregulation of the generation of AMPs in innate immune responses results in increased susceptibility to microbial infections (Ganz, 2003; Porto et al., 2016; Arranz-Trullen et al., 2017; Modi et al., 2017). Amongst the AMP family, some of the important AMPs are human neutrophil peptide 1–3 (HNP1-3), human beta defensin-2 (HBD-2), granulysin, and cathelicidin (LL-37).

We hypothesized that low BMI would diminish the AMPs levels in latent tuberculosis (LTBI) individuals. To study the influence of low BMI on LTBI, we examined the systemic circulating levels, baseline, and antigen stimulated levels of AMPs such as HNP 1-3, granulysin, HBD-2, and cathelicidin (LL-37) in individuals with LTBI and low BMI (LBMI) and compared them with those in individuals with LTBI and normal BMI (NBMI).

## Methods

### Ethics Statement

All individuals were examined as part of a clinical protocol approved by the Institutional Review Board of the National Institute of Research in Tuberculosis (approval nos. NCT00375583 and NCT00001230), and informed written consent was obtained from all individuals.

### Study Population

We recruited 132 participants with latent TB infection: 44 with LBMI, 44 with NBMI, and 44 healthy controls (HC) (Table 1) in Kanchipuram District, Tamil Nadu, South India. All participants were aged between 18 and 65 years and were enrolled consecutively. Participants were screened in a community wide study and were not contacts of TB cases.

**Table 1A:** Demographic profile of the study population.

**Study demographics**	**LBMI**	**NBMI**	**HC**
	***n* = 44**	***n* = 44**	***n* = 44**
M/F	24/20	21/23	24/20
Age	39 (24–60)	41 (24–60)	41 (23–59)
Body mass index	15.9 (13.9–17.8)	23 (18.8–24.8)	22 (18.7–24.0)

**Table 1B:** Biochemical parameters of the study population.

**Biochemical parameters**	**LBMI**	**NBMI**	**HC**	***p*-value**
Albumin (g/dl)	2.8 (2.6–3.2)	4.2 (3.7–4.6)	4.1 (3.8–4.5)	0.0335
Random Blood Glucose (mg/dl)	89.4 (65–106)	94.7 (73–178)	95 (85–160)	0.8281
HbA1c (%)	5.3 (4.7–6.1)	5.8 (4.7–6.0)	5.7 (4.5–6.0)	0.8463
Urea (mg/dl)	21.7 (8–36)	21.3 (11–37)	18.5 (9–22)	0.8316
Creatinine (mg/dl)	0.76 (0.2–1)	0.74 (0.6–1)	0.64 (0.5–0.9)	0.6181
ALT (U/L)	18.7 (8–93)	21.8 (7–45)	20.5 (8–50)	0.7938
AST (U/L)	23.8 (13–68)	25.2 (13–54)	24.2 (8–51)	0.6826

*The values represent geometric mean and range except for gender and age (which is median and range). LBMI refers to individuals with low BMI with LTBI, NBMI refers to individuals with normal BMI with LTBI*.

### Diagnosis of LTBI

LTBI was diagnosed as those who were positive for both tuberculin skin test (TST) and QFT, with no symptoms or signs of active TB, no history of previous TB, and normal chest radiographs. TST was performed using 2 tuberculin units of tuberculin purified protein derivative (PPD) RT 23 SSI (Serum Statens Institute). A positive skin test was defined as an induration of at least 12 mm in diameter, based on the previously defined cutoff norms for South India (13). The recruited participants tested negative for diabetes, HIV, and parasitic infection. Healthy control (HC) individuals were negative for QFT and TST, asymptomatic, and with normal chest radiographs.

### Sample Collection

5 ml of blood was obtained from LBMI and NBMI study participants and 2 ml from HC individuals, which was used for circulating levels alone. 1 ml of whole blood was incubated *in vitro* with either no antigen (NIL) or a cocktail of TB antigens (ESAT-6, CFP-10, TB 7.7) (TB Ag) in Quantiferon TB gold in Tube test (QFT) tubes. This was done at 37°C for 18 hrs and supernatants were collected. The rest of the whole blood was spun down, and plasma was separated. Plasma and the QFT supernatant samples were collected from the same individuals. The samples were stored at −80°C until further use.

### QFT ELISA

QFT was done following manufacturer's instructions (Qiagen, Valencia, USA). Conjugate and supernatant samples and standards were added to the corresponding wells in duplicates and the plate incubated for 120 min at room temperature. After washing, the enzyme substrate was added and incubated for 30 min at room temperature. Finally, stop solution was added and the plate read at 450 nm filter and with a 620–650 nm reference filter. OD values are used to calculate results for IFNγ values in International Units. QFT was performed during the initial screening.

### Measurement of BMI and Biochemical Parameters

Anthropometric measurements, including height, weight, and waist circumference, and bio- chemical parameters, including plasma glucose, serum albumin, urea, creatinine, alanine aminotransferase (ALT), aspartate aminotransferase (AST), and HbA1c levels were obtained using normalized techniques. Low and normal BMIs were termed based on the American Heart Association/American College of Cardiology guidelines (LBMI, < 18.5 kg/m2; and NBMI, between 18.5 and 24.9 kg/m2). Additionally, undernutrition was confirmed by low serum albumin (<3.4 g/dl) in all the low BMI individuals. The presence of intestinal parasitic infection was ruled out by performing stool microscopy. TropBio enzyme-linked immunosorbent assay (ELISA) method was used to exclude filarial infection.

### ELISA

Circulating levels of HNP 1–3 and HBD-2 were measured using Mybiosource ELISA kits. Cathelicidin (LL-37) was measured using Hycult biotech and Granulysin was measured using Duo-set ELISA Development System (R&D Systems) using plasma samples.

The baseline (unstimulated) or TB antigen whole blood supernatants from QFT tubes were then used to measure the levels of HNP 1-3, Granulysin, HBD-2, and Cathelicidin (LL-37). TB antigen stimulated levels are shown as net levels with baseline (unstimulated) levels subtracted. The lowest detection limits were as follows: HBD-2, 15.6 pg/ml; HNP1-3, 0.625 ng/ml; LL-37, 0.1 ng/ml, Granulysin, 15.625 pg/ml.

### Statistical Analysis

Sample size calculation was done to detect a significant difference (*p* < 0.05) among the anti-microbial peptides based on preliminary analysis between the two groups. We determined that we needed 44 individuals in each group to detect this difference with a power of 90% and a Type I error of 5%. Geometric means (GM) were used for measurements of central tendency. Statistically significant differences between the three groups were analyzed using the Kruskal- Wallis test with Dunn's *post-hoc* for multiple comparisons. Mann–Whitney *U*-test with Holm's correction for multiple comparisons was used between two groups. Statistical significant correlations were analyzed using Spearman Rank Correlation. Data entry was made using Microsoft Excel. Analyses were performed using GRAPHPAD PRISM Version 6 (GraphPad, San Diego, CA).

## Results

### Study Population Characteristics

The baseline demographics of the study population are shown in Table 1. There were no differences in age or sex between the groups. The two groups did not differ significantly in the levels of random blood glucose, HbA1c, urea, creatinine, ALT, and AST levels, with the exception of serum albumin levels (*p* = 0.0365).

### Low BMI Is Associated With Diminished Circulating Levels of HNP1-3, Granulysin, HBD-2 and Cathelicidin

**Figure 1:**
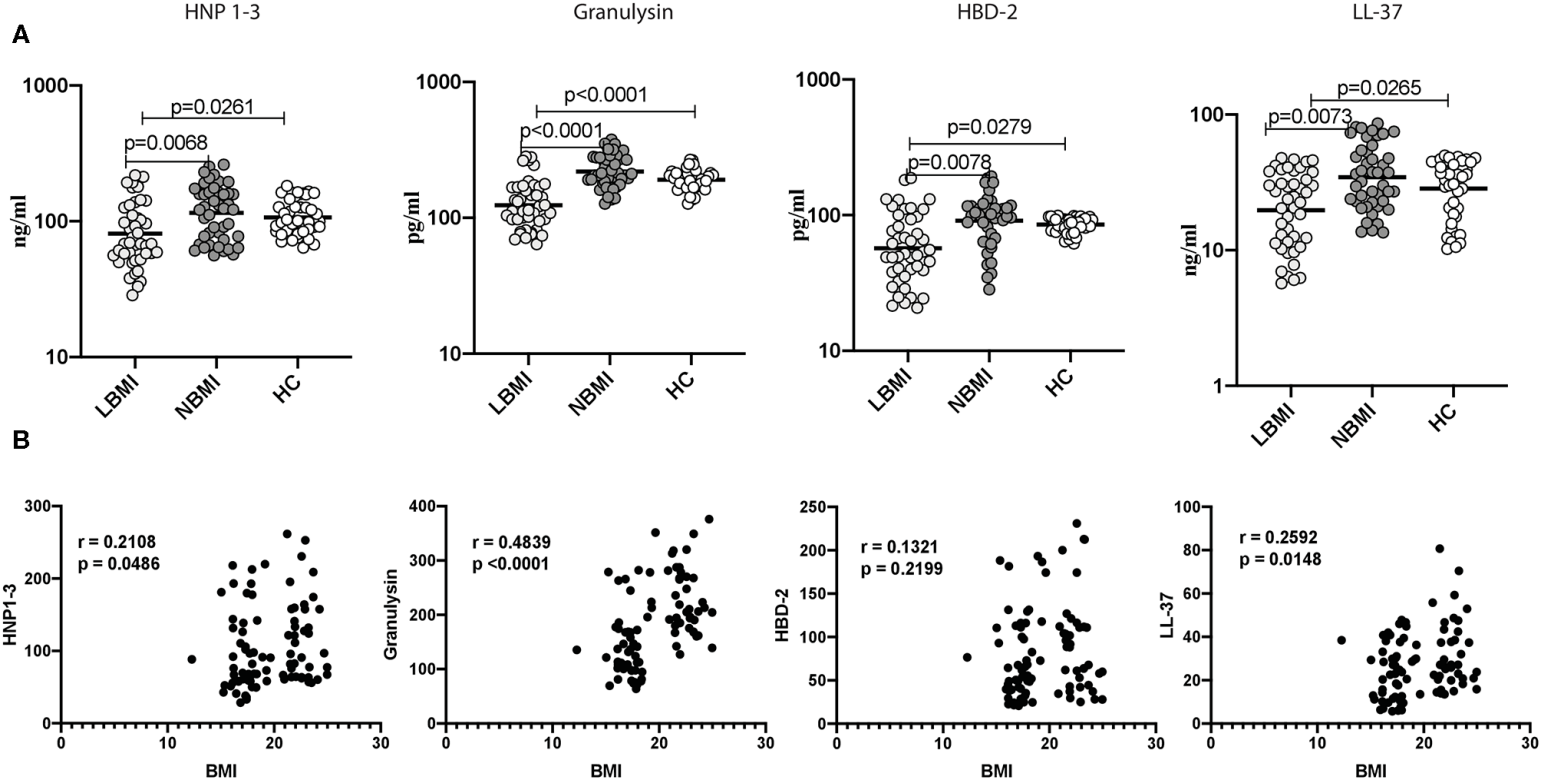
Low BMI is associated with diminished circulating levels of HNP1-3, granulysin, HBD-2 and cathelicidin. **(A)** The plasma levels of HNP1-3, granulysin, HBD-2, and cathelicidin (LL37) were measured by ELISA in LTBI-LBMI (*n* = 44), LTBI-NBMI (*n* = 44), and HC (*n* = 44) individuals. The data are depicted as scatter plots and each circle represents a single person. (open circle–LTBI with LBMI and light gray–LTBI with NBMI and dark gray- LTBI with HBMI). *P*-values were calculated using the Kruskal Wallis test with Dunn's multiple comparison test. **(B)** Relationship between the levels of HNP1-3, granulysin, HBD-2, and cathelicidin (LL37) and BMI in LTBI individuals. The relationship between the systemic levels of HNP1-3 and granulysin, HBD-2 and cathelicidin (LL37) and BMI were examined in all LTBI (*n* = 88) individuals. *p* and *r*-values were determined by Spearman rank correlation at 95% confidence intervals.

To determine the systemic levels of circulating AMPs in LBMI, NBMI, and HC, we measured the circulating levels of HNP 1-3, granulysin, HBD-2, and cathelicidin (LL-37) in LBMI and NBMI individuals with coexistent LTBI and in HC individuals. As can be seen in Figure 1A, the circulating levels of HNP 1-3 (Geometric Mean (GM) of 81 ng/ml in LBMI, 105 ng/ml in NBMI; 106.7 pg/ml in HC *p* = 0.0032), Granulysin (GM of 124 pg/ml in LBMI; 219 pg/ml in NBMI; 191 pg/ml in HC *p* < 0.0001), HBD-2 (GM of 57 pg/ml in LBMI; 83 pg/ml in NBMI; 85.37 pg/ml in HC *p* < 0.0001) and cathelicidin (LL-37) (GM of 20 ng/ml in LBMI; 28 ng/ml in NBMI; 28.48 ng/ml in HC *p* = 0.0008) were significantly lower in LBMI individuals. Next, we wanted to determine the relationship between the circulating levels of HNP 1-3, granulysin, HBD-2, and LL-37 and BMI. As can be seen in Figure 1B, the circulating levels of HNP1-3, granulysin, and LL-37 exhibited significant positive correlation with BMI. In contrast, HBD-2 levels did not show any significant correlation with BMI. Thus, LBMI is associated with diminished systemic levels of circulating AMPs, which have a significant positive correlation with BMI in LTBI individuals.

### Low BMI Is Associated With Diminished Baseline (Unstimulated) Levels of HNP1-3 and Granulysin

**Figure 2:**
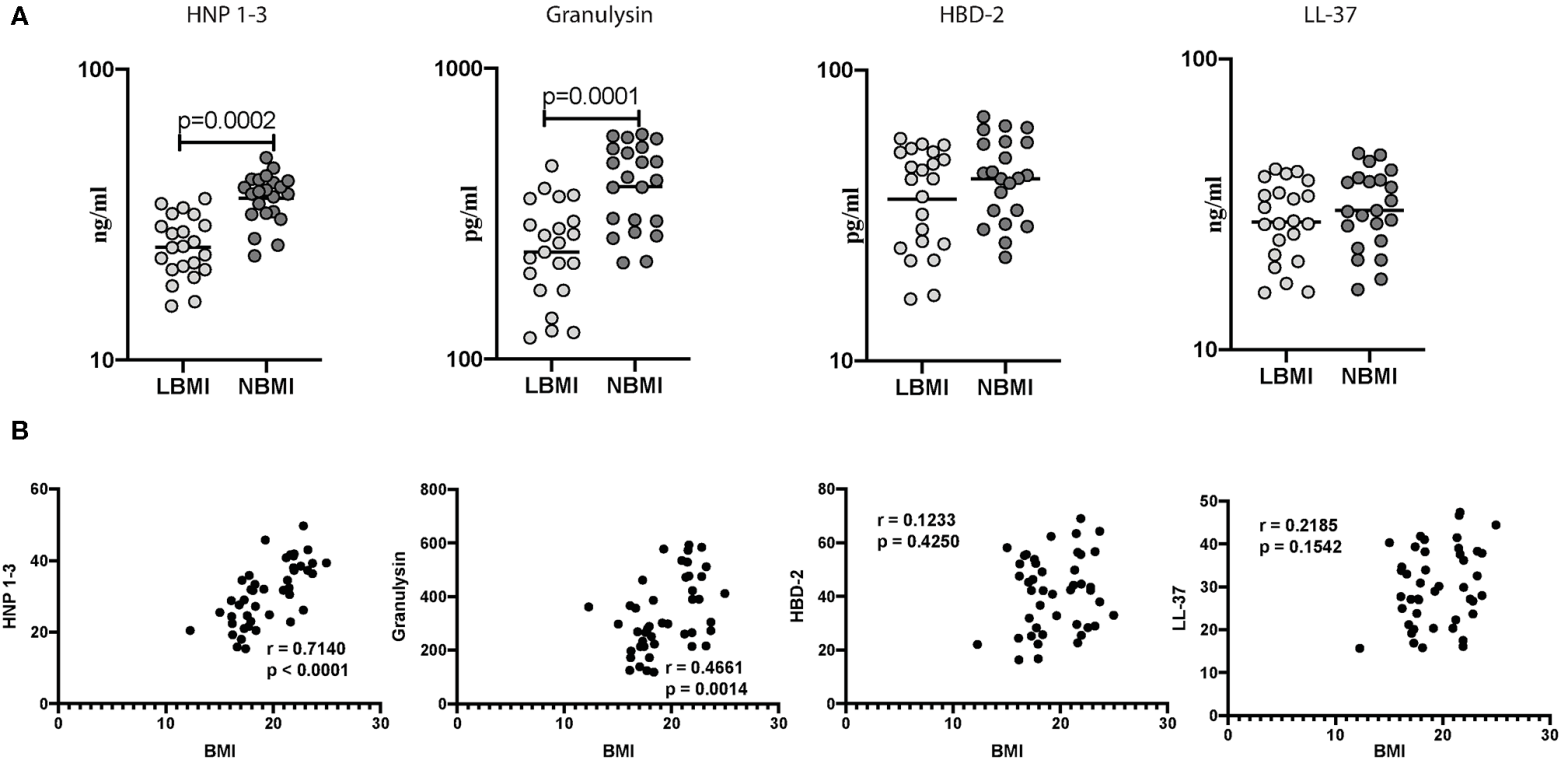
Low BMI is associated with diminished baseline (unstimulated) levels of HNP1-3 and granulysin. **(A)** The baseline (unstimulated) levels of HNP1-3, granulysin, HBD-2, and cathelicidin (LL37) were measured by ELISA in LTBI-LBMI (*n* = 44) and LTBI-NBMI (*n* = 44) individuals. The data are depicted as scatter plots and each circle represents a single person. Mann–Whitney *U*-test with Holms correction for multiple comparisons were done to calculate *p*-values. **(B)** Relationship between the levels of HNP1-3, granulysin, HBD-2, and cathelicidin (LL37) and BMI in LTBI individuals. The relationship between the baseline (unstimulated) levels of HNP1-3, granulysin, HBD-2, and cathelicidin (LL37) and BMI were examined in all LTBI (*n* = 44) individuals. *p* and *r*-values were determined by Spearman rank correlation at 95% confidence intervals.

Next, we wanted to determine the influence of BMI on unstimulated levels of AMPs, and to this end, we measured the baseline (unstimulated) levels of HNP 1-3, Granulysin, HBD-2, and cathelicidin (LL-37) in LBMI and NBMI individuals with coexistent LTBI. As can be seen in Figure 2A, the baseline (unstimulated) levels of HNP 1-3 (GM of 24 ng/ml in LBMI vs. 36 ng/ml in NBMI; *p* < 0.0001) and granulysin (GM of 234 pg/ml in LBMI vs. 392 pg/ml in NBMI; *p* < 0.0001) were significantly lower in LBMI individuals. The levels of HBD-2 and cathelicidin (LL-37) did not differ significantly between the two groups. Next, we wanted to determine the relationship between the baseline (unstimulated) levels of HNP 1-3, granulysin, HBD-2, and LL-37 and BMI. As can be seen in Figure 2B, the baseline (unstimulated) levels of HNP1-3 and granulysin exhibited a significant positive correlation with BMI. In contrast, HBD-2 and cathelicidin (LL-37) levels did not show any significant correlation with BMI. Thus, LBMI is associated with diminished baseline levels of certain AMPs and HNP 1-3 and granulysin exhibited a significant positive correlation with BMI in LTBI individuals.

### Low BMI Is Associated With Diminished Mycobacterial-Antigen Stimulated Levels of HNP1-3, Granulysin, HBD-2 and Cathelicidin

**Figure 3:**
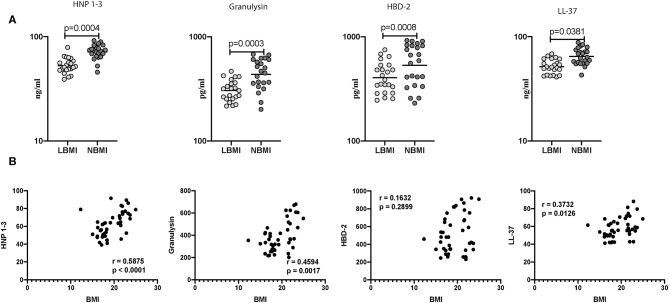
Low BMI is associated with diminished mycobacterial-antigen stimulated levels of HNP1-3, granulysin, HBD-2 and cathelicidin. **(A)** The mycobacterial-antigen stimulated levels of HNP1-3, granulysin, HBD-2, and cathelicidin (LL37) were measured by ELISA in LTBI-LBMI (*n* = 44) and LTBI-NBMI (*n* = 44) individuals. The data are depicted as scatter plots and each circle represents a single person. Mann–Whitney *U*-test with Holms correction for multiple comparisons were done to calculate *p*-values. **(B)** Relationship between the levels of HNP1-3, granulysin, HBD-2, and cathelicidin (LL37) and BMI in LTBI individuals. The relationship between the mycobacterial-antigen stimulated levels of HNP1-3 and granulysin, HBD-2 and cathelicidin (LL37) and BMI were examined in all LTBI (*n* = 44) individuals. *p* and *r*-values were determined by Spearman rank correlation test at 95% confidence intervals.

Subsequently, we wanted to determine the influence of BMI on mycobacterial stimulated levels of AMPs, and to this end, we measured the antigen stimulated levels of HNP 1-3, granulysin, HBD-2, and cathelicidin (LL-37) in LBMI and NBMI individuals with coexistent LTBI. As can be seen in Figure 3A, the antigen stimulated levels of HNP 1-3 (GM of 53 ng/ml in LBMI vs. 71 ng/ml in NBMI; *p* < 0.0001), granulysin (GM of 305 pg/ml in LBMI vs. 436 pg/ml in NBMI; *p* = 0.0004), HBD-2 (GM of 404 pg/ml in LBMI vs. 534 pg/ml in NBMI; *p* = 0.0381), and cathelicidin (LL-37) (GM of 52 ng/ml in LBMI vs. 65 ng/ml in NBMI; *p* < 0.0001) were significantly lower in LBMI individuals. Next, we wanted to examine the relationship between the antigen stimulated levels of HNP 1-3, granulysin, HBD-2, and LL-37 and BMI. As can be seen in Figure 3B, the antigen stimulated levels of HNP1-3, granulysin, and cathelicidin (LL-37) exhibited a significant positive correlation with BMI. HBD-2 levels did not show any significant correlation with BMI. Thus, LBMI is associated with diminished antigen stimulated levels of AMPs, which have a significant positive correlation (with the exception of HBD-2) with BMI in LTBI individuals.

### Low BMI Is Associated With Diminished IFNγ Levels at Baseline (Unstimulated) and Following TB-Antigen Stimulation

**Figure 4:**
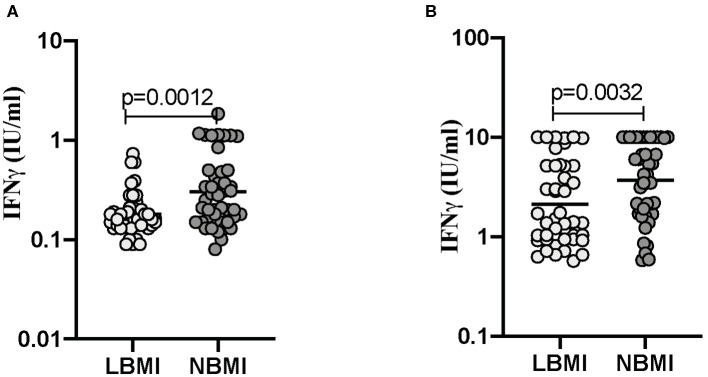
Low BMI is associated with diminished IFNγ levels at baseline (unstimulated) and following TB-antigen stimulation. The baseline (unstimulated) and TB - antigen stimulated IFNγ levels were measured by QFT ELISA in LTBI-LBMI (*n* = 44) and LTBI-NBMI (*n* = 44) individuals. The data are depicted as scatter plots and each circle represents a single person. Mann–Whitney *U*-test was done to calculate *p*-values. **(A)** Baseline (unstimulated). **(B)** TB-Ag stimulation.

Next, we wanted to determine the influence of BMI on the release of IFNγ levels at baseline (unstimulated) and following TB-antigen stimulation in LBMI and NBMI individuals with concomitant LTBI. As can be seen in Figure 4, the baseline (unstimulated) levels of IFNγ (GM of 0.1804 IU/ml in LBMI vs. 0.3042 IU/ml in NBMI; *p* = 0.0012) was significantly lower in LBMI individuals. Upon TB antigen stimulation, the levels of IFNγ (GM of 2.135 IU/ml in LBMI vs. 3.712 IU/ml in NBMI; *p* = 0.0032) was also significantly lower in LBMI individuals. Thus, LBMI is associated with diminished baseline (unstimulated) and antigen stimulated levels of IFNγ.

## Discussion

AMPs have the capacity to modulate the innate immune responses of the host and thus indirectly promote pathogen clearance (Hancock and Sahl, 2006; Yeung et al., 2011). Many AMPs destroy pathogens by disrupting the physical integrity of the microbial membrane and/or by translocating across the membrane into the cytoplasm of bacteria to act on intracellular targets (Hancock and Sahl, 2006). In addition to a direct antimicrobial effect, AMPs also have numerous immunomodulatory properties, like modulation of cytokine and chemokine expression and activation of leukocytes (Sorensen et al., 2008; Fjell et al., 2011; Hilchie et al., 2013; Mansour et al., 2014). AMPs display high antibacterial activity but low immunogenicity, and hence are promising anti-mycobacterial therapeutic agents (Mendez-Samperio, 2008).

Neutrophils may mediate innate immune resistance against *M. tb* by inducing bactericidal activity and conferring protection at the early stages of mycobacterial infection (Sharma et al., 2000; Barrios-Payan et al., 2006). Sharma et al. showed that after injection with HNP-1, the mice exhibited clearance of *M.tb* from the lungs, liver, and spleen (Sharma et al., 2001). Another study by Martineau et al. demonstrated that neutrophils play a role in innate immunity against *M.tb*, an activity linked with HNP1-3 (Martineau et al., 2007). Our data reveals that HNP1-3 levels are significantly diminished in systemic circulation, and after baseline or mycobacterial antigen stimulation in LTBI-LBMI compared to LTBI-NBMI group, and positively correlate with BMI. The deficiency of HNP1-3 could lead to the impairment in airway defense function and acquired immunity, which in turn decreases the ability of the host to remove the invading pathogen completely.

Granulysin can kill *M.tb* by altering the membrane integrity and directly eliminates extracellular and intracellular bacteria in the presence of perforin by triggering osmotic shock and provoking apoptosis (Clayberger and Krensky, 2003). Studies have shown that granulysin levels are decreased in adult and pediatric TB patients. Thuong et al. showed that IGRA positive individuals had significantly lower serum granulysin when compared with IGRA negative individuals. In addition, they showed that low serum granulysin concentrations were significantly associated with IGRA status (Thuong et al., 2016). In agreement with these studies, our data showed both systemic and antigen stimulated granulysin levels were diminished in LTBI-LBMI individuals when compared with NBMI. In addition, the levels of granulysin correlated positively with BMI. Granulysin levels may depend upon the nutrition and health status in individuals and the persistence of clinical disease was evidenced to be associated with deficient expression of granulysin at the local site of TB infection (Pitabut et al., 2013), (Andersson J, Infect Immun. 2007). Low granulysin levels in TB may be caused by rapid consumption due to ongoing effector immune response, or due to the reduction of T cell subsets dedicated to its production (Sahiratmadja et al., 2007; Pitabut et al., 2011).

HBD-2 plays a role in immunity against *M.tb* (Kisich et al., 2001) mainly through its chemotactic effects on immature dendritic cells and memory lymphocytes (Yang et al., 1999). The expression of HBD-2 by human macrophages can be triggered by *M.tb* (Zhao et al., 2016). The expression and secretion of defensins are stimulated by pathogen-associated molecular patterns (PAMPs), such as LPS, by TLR (Pivarcsi et al., 2005). This initiates MAPK- or NF-κB-dependent cascades that end in a pro-inflammatory response involving the secretion of cytokines, chemokines, and defensins (Mendez-Samperio et al., 2006). *In vitro* studies suggest that HBD-2 is involved in reducing *M. tb* growth, and the combination of HNP-1 with anti-tuberculosis drugs significantly reduces the mycobacterial load (Mendez-Samperio et al., 2008). Our study reveals that HBD-2 levels are present at significantly diminished levels in both systemic and upon antigen stimulation in the LTBI-LBMI group compared to the LTBI-NBMI group and did not exhibit any significant relationship with BMI. Defensins could be important as a component part of the protection against human tuberculosis (Rivas-Santiago et al., 2005; Dong et al., 2016). In our study, the diminished levels of defensin could lead to the impairment of protective immunity.

Cathelicidin (LL-37) is considered to be a key molecule for the control of TB and it is secreted by neutrophils, monocytes, mast cells, and epithelial cells. *In vitro* studies have clearly determined a key role of cathelicidin in the antimicrobial activity of human macrophages to *M.tb* (Liu and Modlin, 2008). LL-37 participates in the recruitment of T-cells to the site of infection and exhibits various immunomodulatory and antimicrobial activities (Torres-Juarez et al., 2015; Arranz-Trullen et al., 2017). A study by Martineau et al. exhibited that LL-37, along with vitamin-D, could play a role in phagocyte defense against *M.tb*. However, the effects of substantial concentrations of LL-37 on *M.tb* are moderate (Martineau et al., 2007). In corroboration with previous studies, our data reveals that LTBI-LBMI individuals exhibited diminished levels of LL-37 which in turn could induce the impairment of cellular level mechanisms and render LTBI-LBMI individuals more susceptible to active TB disease.

Our data provides an understanding of the influence of nutrition on the pathogenesis of TB. To our knowledge, this is the first study to examine the impact of low BMI on AMPs in the context of LTBI. Our data on AMPs suggest that diminished levels of AMPs is a distinctive feature of LTBI-LBMI individuals. Although our data do not provide any mechanistic conclusions, our study provides important preliminary evidence for a major relationship between LBMI and AMPs in a chronic infection. We postulate that low BMI might directly impact the production of AMPs from both hematopoietic and non-hematopoietic cells and thus lower the levels of AMPs in circulation, especially since the LBMI levels are lower than those in HC. Our study suffers from the limitation of not being a longitudinal study, of not measuring the mitogenic stimulation, or non-specific antigen stimulation of cells. Longitudinal studies of low BMI individuals with LTBI should help elucidate the mechanism by which LBMI promotes the progression from latent to active infection. In conclusion, by studying AMPs responses at systemic circulation, baseline, and antigen stimulated in LBMI individuals with LTBI, our study offers the direct relationship of AMPs in low BMI and LTBI individuals. A previous study by Hang NT et al. has shown that a BMI which is lower than 16.0 exhibited an association with IGRA negativity (Hang et al., 2011). However, we found no correlation with BMI and IFNγ values in our study.

LBMI was associated with diminished systemic levels of HNP1-3, granulysin, HBD-2, and cathelicidin. At baseline, HNP1-3 and granulysin levels were diminished and, upon mycobacterial antigen stimulation, the levels of HNP1-3, granulysin, and HBD-2. Thus, our data, exhibits that coexistent LBMI in LTBI is described by the diminished levels of HNP1-3, granulysin, HBD-2, and cathelicidin.

## Data Availability Statement

The data generated for this study can be found inside the article.

## Ethics Statement

The studies involving human participants were reviewed and approved by National Institute of Research in Tuberculosis (approval nos. NCT00375583 and NCT00001230). The patients/participants provided their written informed consent to participate in this study.

## Author Contributions

SB and AR conceived and planned the experiments and wrote the manuscript. AR and SM executed the experiments. AR analyzed the data. CD provided patient samples. All authors contributed to manuscript revision, read, and approved the submitted version.

## Conflict of Interest

The authors declare that the research was conducted in the absence of any commercial or financial relationships that could be construed as a potential conflict of interest.
